# Microvalve array fabrication using selective PDMS (polydimethylsiloxane) bonding through Perfluorooctyl-trichlorosilane passivation for long-term space exploration

**DOI:** 10.1038/s41598-022-16574-9

**Published:** 2022-07-20

**Authors:** Zachary Estlack, Jungkyu Kim

**Affiliations:** Department of Mechanical Engineering, University of Utah, Salt Lake City, UT 84112 USA

**Keywords:** Biomedical engineering, Chemical engineering

## Abstract

To improve the versatility and robustness of microfluidic analytical devices for space exploration, a programmable microfluidic array (PMA) has been implemented to support a variety of missions. When designing a PMA, normally closed valves are advantageous to avoid cross contamination and leaking. However, a stable fabrication method is required to prevent these valves from sticking and bonding over time. This work presents how polydimethylsiloxane (PDMS) can be bonded selectively using chemical passivation to overcome PDMS sticking issue during long-term space exploration. First, on a PDMS stamp, the vaporized perfluorooctyl-trichlorosilane (PFTCS) are deposited under − 80 kPa and 150 °C conditions. The PFTCS was then transferred onto PDMS or glass substrates by controlling temperature and time and 15 min at 150 °C provides the optimal PFTCS transfer for selective bonding. With these characterized parameters, we successfully demonstrated the fabrication of PMA to support long-term space missions. To estimate the stability of the stamped PFTCS, a PMA has been tested regularly for three years and no stiction or performance alteration was observed. A flight test has been done with a Cessaroni L1395 rocket for high g-force and vibration test and there is no difference on PMA performance after exposure of launch and landing conditions. This work shows promise as a simple and robust technique that will expand the stability and capability of PMA for space exploration.

## Introduction

Microfluidic analytical instruments for space exploration have been developed to determine chemical compositions from small soil or particle samples^1–5^. However, to be compatible for broader missions, programmability and durability requires further development. A programmable microfluidic array (PMA) is designed to achieve autonomous fluidic manipulation such as pulling, pushing, mixing, and fluid distribution with high precision. The design of the microvalve and the operational parameters can be defined to achieve desired dispensing volume and flow rate. PMA have been demonstrated for programmed sample preparation, fluorometric assays, and biosensing to show the versatility using a normally closed microvalves^4,6–12^. Typical normally closed valves have a gate structure either on the flexible membrane side or the microchannel side to block flow without actuation^12–14^. Even though this is an excellent aspect for fluidic control, PMA fabrication requires selective PDMS bonding procedures to minimize valve sticking issue after plasma exposure^15^. Furthermore, PDMS can weakly bond to glass after long term contact. Empirical evidence from PMA produced in our lab implies that if valves rest longer than 6 months, the gates in microvalves stick to glass and PDMS substrates. The typical timeline to reach target planets such as Mars, Europa, and Enceladus are about seven months^16^, five years^17^, and seven years^18^ of travel away, respectively, and thus, to use the PMA for exploring these planets, the microvalve stability issue must be resolved to obtain expected PMA performance.

Typical PMAs are fabricated using a soft-lithography technique with polydimethylsiloxane (PDMS)^19,20^ and packaged by oxygen plasma that treats all exposed surfaces^19,21,22^. Some selective bonding is possible through manual application of a passivating chemical, manually blocking areas, or treating the surface with untreated PDMS^23,24^. However, these are limited in scope to fabricate 3D structures instead of selective bonding with complex microfabricated stamp molds without investigating any long-term effect and stability^23,24^.

Chemical treatment has been done using various silanes to alter surface properties on PDMS microfluidic devices. Among these, Perfluorooctyl-trichlorosilane (PFTCS) is often used to form super-hydrophobic surface^23,25–28^ as it easily deposits on surfaces due to its low vapor pressure. PFTCS also forms stable layers on hydroxylated surfaces, like the surface of PDMS or glass after oxygen plasma treatment, through a condensation reaction^23,29^. While manual PFTCS passivation without any patterning methods, like manual liquid application, can be used for low precision fabrication, actuatable devices with high precision require a scalable selective bonding method.

In this work, we present a simple, precise, and mechanically and chemically resilient technique for selective PDMS bonding to enhance the stability of PMA for long term space missions. We investigate vacuum level, temperature, and time associated with PFTCS deposition to obtain precise PFTCS layer on a PDMS stamp, and the PFTCS transfer between the stamp and a substrate influenced by time, temperature, and the substrate material. By determining detailed physicochemical parameters, we were able to demonstrate faultless PMA fabrication without any sticking issue. We fabricate two example devices with this procedure and test them to demonstrate usability and long-term stability for future space exploration. With all characterization results, this fabrication method can easily be expanded beyond the devices presented in this work for high density microvalve array, stretchable MEMS devices with wafer-scale, and other research fields where selective bonding with long-term stability is required.

## Materials and methods

### PFTCS deposition

**Figure 1:**
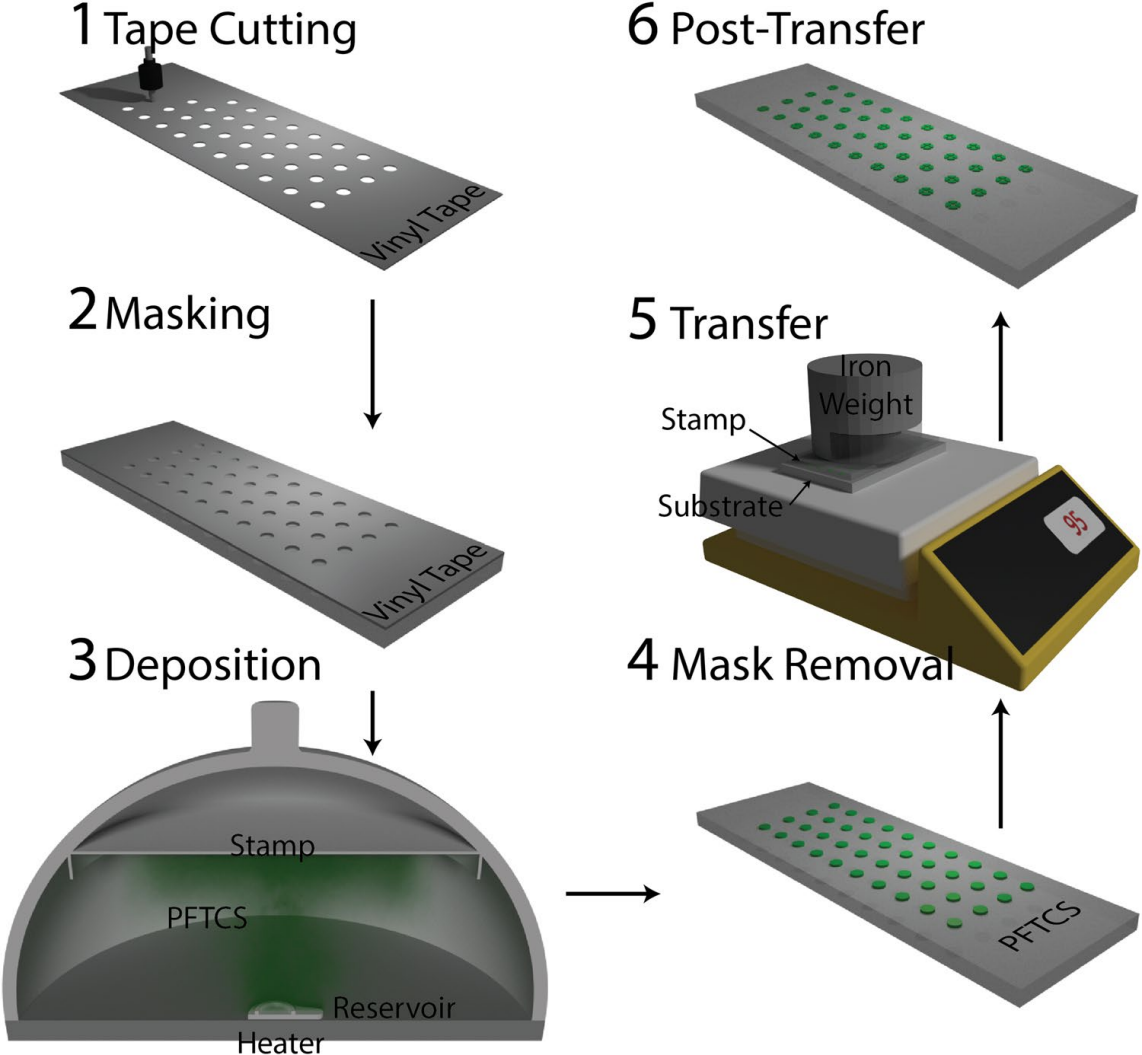
Overview of the deposition and stamping procedure. (1) A vinyl tape mask is cut based on the desired selective bonding pattern. (2) This mask is applied to a PDMS slab that will act as the stamp. (3) Deposition of PFTCS occurs in a low-cost vacuum desiccator. (4) The tape mask is removed from the stamp, leaving the PFTCS only in the exposed area. (5) Heat and force are used to transfer the PFTCS from the stamp to the desired substrate. (6) After transfer, the stamp shows apparent signs of transfer to the substrate for easy quality assurance checks.

As described in Fig. 1, a deposition mask is cut (Roland CAMM-1 Servo GX-24) from vinyl tape (Gerber; Tolland, CT) and applied to a piece of bulk PDMS (Stockwell Elastomerics; Philadelphia, PA) for step 1 and 2. This method allows for 300 µm feature resolution. However, a mask could similarly be created using photolithography resulting in micron scale features. A vacuum chamber (Fisher Scientific, PA) had a 5-inch petri dish lid secured to the top with double-sided tape on it in order to provide a mounting location for the stamps. The stamp was placed in the middle of the top of the chamber, and the PFTCS (Sigma Aldrich, MO USA) was removed from a desiccator for use. A heat block was heated for at least two hours before being placed in the chamber with a microtube top containing PFTCS. In order to start deposition, 100 µL of PFTCS was added to the microtube top, and vacuum is applied. The vacuum level in the chamber was monitored, and the chamber’s valve was closed to isolate it once the required vacuum level was reached. After the desired deposition time, the chamber vacuum was released to obtain a PFTCS deposited stamp described in steps 3 and 4 in Fig. 1.

### Stamping

Stamping was done onto two substrates, glass and PDMS with a cross feature. Prior to stamping, each substrate was cleaned with Acetone (Glass only), IPA, and DI water (All substrates) before drying with N2 gas. A jig was then used to mark the underside of the glass slides to specify where the PFTCS was to be stamped. Each substrate was placed on a hotplate set to the desired temperature for that experiment and allowed to settle at a steady-state temperature, ~ 15 min. The substrates were briefly removed from the hotplate, and the stamps were aligned before being pressed on. Alignment was done using markings on the glass or the PDMS crosses as reference points. These reference points were lined up with the opaque PFTCS deposition on the stamp itself manually. As shown in step 5 of Fig. 1, after alignment, the substrate-stamp combination was moved back to the hotplate, and a 3 kg cylindrical weight was placed on top, corresponding to 6.5 kPa or pressure. At the end of each time step (1,15,30, and 60 min), the stamp was removed from the substrate and stored for measurement along with the featured PDMS, if necessary, leaving the stamp with the residual PFTCS pattern shown in step 6 of Fig. 1.

### Characterization of PFTCS deposition thickness and stamping transfer

All thickness measurements were completed using an Olympus LEXT OLS5000 (Olympus, Japan). PFTCS film thickness and height variation were determined using the Olympus software step utility, comparing the average height between two regions of interest in the profile. Roughness measurement was completed by obtaining the variation in height across a region of interest set to be the PFTCS deposition area. Transfer uniformity was quantified using ImageJ to select areas of transfer of PFTCS and comparing to the overall possible transfer area. Scanning electron microscope (SEM) measurements were done with a FEI Quanta 600F (Oregon, USA) using the secondary electron detector. Samples were prepared as otherwise detailed in the methods prior to SEM measurement at the Quanta’s low vacuum mode.

### A simple PMA design and fabrication

Figure SI1 shows a simple five microvalve chip design in AutoCAD^12^. This chip was designed to be a simple platform to test selective bonding. The design consisted of four two-way valves and a central four-way valve. The required molds were fabricated using conventional xurography methods using a vinyl cutter and vinyl tape^30^. Two molds for the devices were required, corresponding to the pneumatic layer and fluidic layer of the final device. The fluidic layer must be thin for the valve membrane to function correctly, so the tape pattern was placed on the lid of a petri dish for spin coating. This lid had uncured PDMS (10:1) poured over the top and was spun at 200 rpm for ~ 150-micron thickness. The thickness of the pneumatic layer was not critical, so its pattern was placed inside a petri dish, and uncured PDMS was poured to ~ 3 mm thickness. The two layers were cured at 75 °C for two hours. The cured pneumatic layer was removed from the mold, and the pneumatic inlets were punched. The fluidic layer was removed from its mold by pressing a flexible plastic sheet to the PMDS and peeling the layer from the mold gradually. A second plastic sheet was placed on the other side to prevent dust contamination. Because each mold had features for nine chips, the fluidic layer was cut apart with scissors, and the pneumatic layer was cut with a razor. The fluidic and pneumatic pieces were briefly cleaned with tape before oxygen plasma treatment for permanent bonding^22^. Briefly, matching pneumatic and fluidic elements were placed into a plasma chamber for exposure. After exposure, each was briefly cleaned with N_2_ gas to remove any dust, aligned using the gates as a guide, and then brought into contact. Next, the devices were placed on a hotplate set to 65 °C for 30 min to complete bonding. Last, the fluidic ports were punched, and the chips were wrapped in plastic wrap to avoid contamination during storage.

### PMA stamp mask design and fabrication

In addition to the gate valve chip, a corresponding stamp mask was designed in AutoCAD. The mask had open areas that corresponded to the location of the microvalves of the simple PMA. The mask was cut using the same method previously described, and the unmasked locations were manually pruned. Next, a 3 mm thick, 90 mm diameter PDMS cylinder was cured, and the mask was transferred to this cylinder. The PDMS was cut into individual stamps, and each was stored until deposition.

### Bonding of featured PDMS and glass

The bonding procedures are almost identical for the glass stamped and featured PDMS devices. In the stamped glass case, the glass and four PDMS chips were placed into the oxygen plasma chamber. PFTCS itself is stable under oxygen plasma and testing showed less than 10% decrease in thickness with the exposure parameters used in this work. For the stamped PDMS microvalves, four treated chips and a glass slide were placed in a chamber. In both cases, the glass and PDMS were treated with an oxygen plasma (PE-50 from Plasma Etch, NV) for 30 s at 200mTorr and 80% power. The treated featured PDMS chips were simply bonded to the glass slide, and the untreated PDMS chips were aligned with the marks on the underside of the treated glass to align the features correctly with the transferred PFTCS. After alignment, the assembled chips were placed on a hotplate set to 65 °C for 30 min to complete bonding.

### Testing of simple PMA and stretchable 3D structure

The initial actuation test of the simple PMA was run first by applying vacuum to each microvalve. Once the functionality of each microvalve was confirmed, transfer uniformity was verified by checking for leakage. Nonuniform transfer would lead to poor sealing of the valve due to the small height differences across the gate. Two microvalves were connected to a 50 kPa nitrogen source as a closing pressure^12^, while the other two were connected to a vacuum. Based on the data in Fig. 5C a flow rate of 90 µL/min was used to be well over the peak pressure during regular microvalve pumping operation, and the pressurized valves were monitored for signs of leaking under these conditions.

Additionally, a stretchable ribbon device was fabricated as a separate demonstration of this method. First, a stamp with a deposition mask was prepared and the mask is shown in Fig. SI2. Deposition onto the stamp proceeded as previously discussed with a source temperature of 115 °C and a deposition time of 30 min. Next, a 75 × 25 mm PDMS strip was stretched and secured to a glass slide. The stamp was removed from the chamber and pressed onto the PDMS strip, stamping for 30 min at 150 °C. Last, the stamped PDMS was bonded to the PDMS ribbon design shown in Fig. SI2 and then relaxed, where separation was observed based on the stamped areas.

### Stability and longevity testing of PMA

A larger scale PMA consisting of 25 two- and four-way valves was fabricated using the optimized parameters detailed above and tested for functionality after long term storage and up subject to high numbers of actuations. Briefly, SU-8 molds for both pneumatic and fluidic layers were fabricated using a standard photolithography protocol^4,12^. The fabrication then proceeded as previously described for the 5-valve chip. A stamp was created and applied using the optimized parameters determined before the PDMS was bonded to a glass wafer. A pumping sequence was used to demonstrate the actuation of all microvalves for transporting liquid from an inlet to an outlet. Flow rate measurements were taken using a Sensirion (Switzerland) SLI-1000 flow rate monitor and compared either after long term dry storage or over an extended period of repeated actuation. Furthermore, the PMA was challenged on a rocket launch to be exposed high g-force condition and rocket launch-induced vibration to confirm the stability of PFTCS coating. The flight parameters are included in Table SI1 and a link to view the flight video is also included. A video of the launch is also included with the supplemental info. A VB300 (Extech; Nashua, NH) g-force data logger was used to measure acceleration and vibration during this launch.

## Results and discussion

### PFTCS deposition

First, the vaporization rates of liquid PFTCS were investigated with respect to time to determine reasonable deposition times, as shown in Fig. SI4. This initial test was done at − 80 kPa, which is well over the vapor pressure of PFTCS. Under this pressure level, the vaporization rate of PFTCS started relatively high (7.73 mg/min) and exponentially dropped and reached an equilibrium of the vaporization rate (2.08 mg/min) after 60 min. After these initial evaluations, actual PFTCS deposition on a PDMS stamp was tested with three levels of three different parameters, considering the thermodynamic properties of PFTCS: chamber vacuum (− 5, − 37.5, and − 80 kPa), deposition time (20, 30, and 40 min), and source temperature (85, 115, and 145 °C). For the chamber vacuum, we chose vacuum levels just above and below the vapor pressure of PFTCS (− 35 kPa)^31^ and a reasonable high vacuum level for a laboratory setting. The times were chosen based on the vaporization experiments as they are long enough for PFTCS molecules to be present in the chamber, while being long enough to be within 15% of the steady-state value. The source temperatures were selected to investigate the influence of the PFTCS vaporization to promote deposition rate without damaging the PDMS^32^ and ensure vaporization at the low end^31^. Figure SI4 shows the deposition of PFTCS under these control parameters. From ANOVA testing (n = 3 for all parameters and levels), we found that the most influential parameter among these parameters was the level of the chamber vacuum (α = 0.05). Time and temperature show less impact on overall deposition over pressure, but impact the overall stability of deposition. Thus, the 30 min deposition time and 115 °C source temperature case shows the lowest standard deviation of significant deposition amounts.

**Figure 2:**
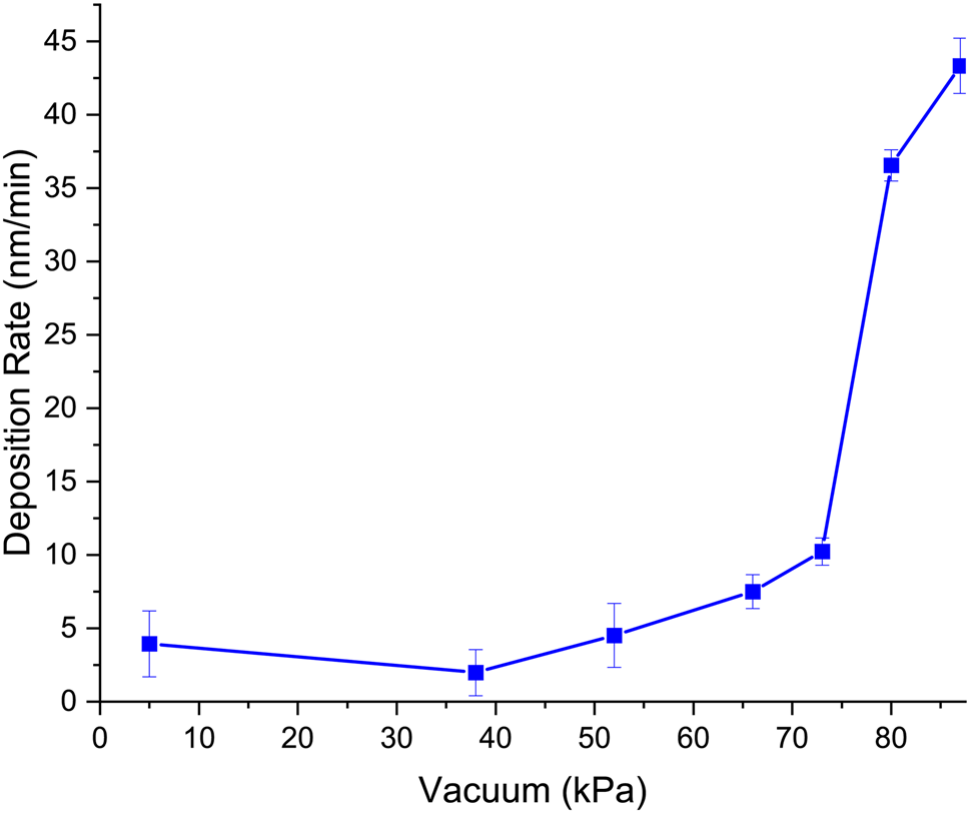
The measured deposition rate of PFTCS depending on vacuum level. All experiments measurements were done after 30 min of deposition and in triplicate. The sharp increase at 73 kPa is due to the vapor pressure of PFTCS and deposition at lower vacuum levels is primarily due to the volatility of the PFTCS.

Further investigation was done with various vacuum pressures to determine the optimal condition for PFTCS deposition presented in Fig. 2. At higher than the PFTCS vapor pressure, the deposition rate increases exponentially between − 73 and − 80 kPa increasing an average of 3.6 nm/min/kPa (36.5 nm/min deposition at − 80 kPa versus 10.23 nm/min deposition at − 73 kPa). However, beyond − 87 kPa vacuum, this deposition rate per vacuums level falls 0.97 nm/min/kPa. At lower than the PFTCS vapor pressure, there is little change in the rate of deposition due to limited PFTCS molecules. Increasing the vacuum greater than that critical vacuum level led to more consistent deposition onto the PDMS stamp surface as more of the vaporized PFTCS molecule filled the chamber. Interestingly, we observed deposition rates increasing exponentially from − 73 to − 80 kPa. Thus, considering the overall deposition rate and consistency, we chose to use − 80 kPa vacuum, 115 °C source temperature, and 30-min deposition time for the remaining experiments.

### Transfer PFTCS on substrates

**Figure 3:**
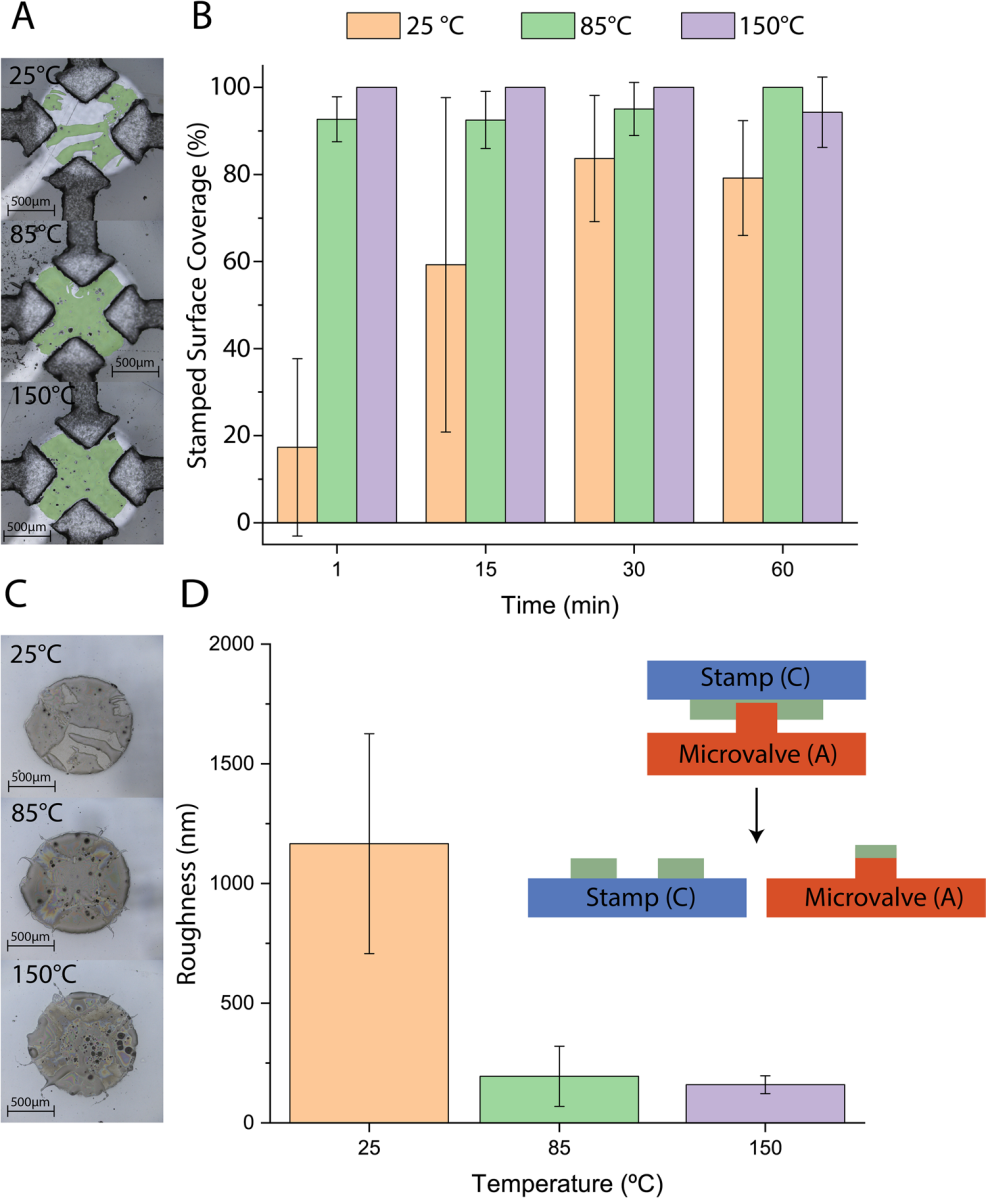
Characterization of PDMS-PDMS stamping to a cross pattern. (**A)** Image of the cross after stamping with PFTCS highlighted in green. (**B**) Surface coverage with respect to stamping time and temperature. (**C**) Image of the corresponding stamps after stamping for 30 min. (**D**) Average RMS height across three locations of each stamp showing the uniformity of transfer. Inset image describes where A and C where obtained. All experiments were performed in triplicate.

To demonstrate PFTCS transfer to a cross-patterned PDMS, a shadow mask with the array of circular window were attached on a PDMS sheet and PFTCS was deposited with the optimal conditions above to create the array of PFTCS spot. This PFTCS was transferred on the cross-patterned PDMS with controlling substrate temperature and stamping time. Figure 3A and B present the transfer of the PFTCS on the cross-patterned PDMS surface with respect to contact time and temperature. We observed that temperature was a critical parameter while time on the transfer amount was very minimal except in the room temperature case. By elevating temperature during the contact, the coverage of PFTCS on the cross pattern is uniform with thin while the room temperature case shows inconsistent PFTCS deposition. This is due to the thermal energy promoting a condensation reaction on both the stamp and substrate surfaces, causing the PFTCS to bind to PDMS surface. Since there is no condensation reaction in the room temperature case, we observed significant variation of PFTCS thickness on both the stamp and the substrate surface.

Transfer area is critical since patchy transfer can lead to partial or complete sticking or an uneven surface, depending on the final application of the substrate. Figure 3A and B show the surface coverage of the corresponding PDMS features and the stamped surface coverage by controlling temperature and stamping time. As indicated with green color in Fig. 3A, a distinct increase in surface coverage can be seen as temperature increases. By performing parametric studies with temperature and stamping time, we were able to determine surface coverage changes. Figure 3B shows that in the case of stamping under room temperature, PFTCS surface coverage increased from 17 to 79% by increasing from 1 to 60 min stamping times and heating the substrate during stamping resulted in greater than 95% of surface coverage regardless of time, likely due to the preheating of the substrate. However, increasing the temperature from 85 to 150 °C did not significantly improve the coverage because perfectly uniform transfer was already possible at the lower temperature. Simulation of the heat transfer during the stamping process described in Fig. SI5 shows that temperatures quickly reach over 57 and 92 °C at the PFTCS interface for the 85 and 150 °C hotplate cases, respectively, due to the preheating of the substrate that took place. The temperatures settle at 65 and 110 °C due to the stamping weight acting as a heatsink. This heatsink action causes continuous heat flux through the stamping interface and the temperature and experimental results show that a condensation reaction is likely occurring, causing layers of the PFTCS to adhere to the crosses instead of the chunks seen in the room temperature case.

Figure 3C and D quantify the roughness of the transfer by reporting the RMS height across three points on each of the stamps. RMS height reports the standard deviation of the height of the film indicating the thickness uniformity of the transferred PFTCS. The RMS height of the stamped surface decreases greatly from 1166 ± 459 nm at 25 °C to 194 ± 125 nm at 85 °C hot plate temperatures, showing the increase in uniformity, but there is only a 20% change between 85 °C and the height of 159.5 ± 37 nm at 150 °C. However, the RMS height at 150 °C does have a much lower standard deviation, indicating the uniformity in the surface across the stamp surface.

Further demonstration has been performed with a glass substrate. Transfer to a bare glass was consistent across all temperatures and times due the abundance of hydroxyl groups on the surface of the glass when compared to untreated PDMS^33,34^. This property promotes the PFTCS adherence to the surface enabling a uniform PFTCS film (Fig. SI6). Regardless of temperature, there was no detectable amount of PFTCS on the stamp and the glass substrate showed uniform thickness when imaged, an average roughness of 350 ± 113 nm across the three images.

### Microvalve array and stretchable 3D structure using the selective PDMS bonding

**Figure 4:**
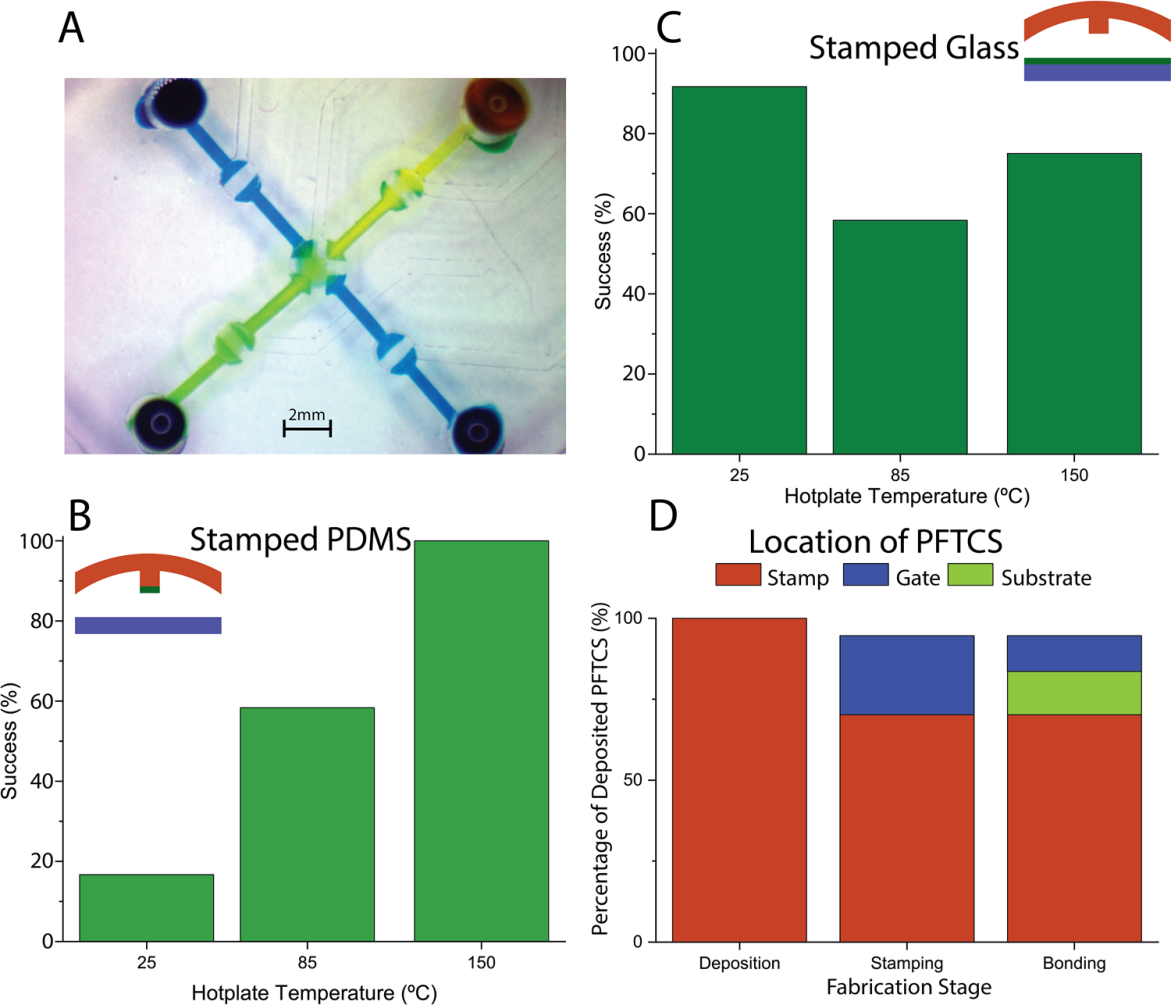
Characterization of transfer of PFTCS from a PDMS stamp to different substrates. (**A**) A simple 5-microvalve chip design. (B-C) The success rate of PFTCS transferred from a PDMS stamp to PDMS features and to glass, respectively. The inset images in (**B**) and (**C**) show the location of the PFTCS (green) with respect to the PDMS (red) and glass (blue). (**D**) PFTCS thickness tracked throughout the fabrication process of a PDMS gate stamped with PFTCS under the optimized fabrication parameters. All results are from 10 of the 5-microvalve chips shown in (**A**).

Further selective bonding was tested for glass and featured PDMS. In practice, the cross pattern transferred to the featured PDMS in the earlier experiment matches the transfer that is required for passivating microvalves. Thus, selective bonding was confirmed by testing both stamped microvalves and the microvalves bonded to stamped glass. Figure 4A presents a simple microvalve array with 5 microvalve network that is used for the experiments and characterization. During microvalve array fabrication, we observed some of the partial or complete failures due to misalignment of the stamp, partially missing the microvalve, or the area the microvalve contacts leading to the valve sticking issue. Overall success rate on a glass or PDMS substrate were estimated. Figure 4B shows the success rate for glass stamped chips. There is no apparent pattern in the success rate as stamping temperature varied changing from 92 to 75% for room temperature and 150 °C stamping, respectively. The glass stamped chips were consistently well-performing due to the consistency of transfer as previously noted. However, alignment with stamped glass was difficult due to the lack of PFTCS visibility, leading to failures due to misalignment of the microvalves. In general, stamping onto a bare glass substrate is not recommended since deposition directly onto glass leverages the opacity of the deposited PFTCS for simple alignment. For the featured PDMS substrate shown in Fig. 4C, a noticeable trend towards the need for higher transfer temperature is seen, 17–100% success as temperature is increased. As previously discussed, low temperature stamping leads to poor transfer to features and caused widespread sticking issues across the chip. Thus, least 100 °C should be used during stamping of featured PDMS to cause a condensation reaction, resulting in high transfer uniformity and device functionality.

In addition, further characterization has been performed to understand the stability of the selective bonding of microvalves using SEM. After checking the gate of microvalve as well as the contacting surface, we found that PFTCS presents on both sides. As indicated in Fig. SI7C, the PFTCS layers formed on both surfaces with roughly half of the PFTCS on the gate that enables the microvalve to be operated without any sticking issues for long period of time. Figure 4D gives a breakdown of the distribution of PFTCS throughout the whole fabrication process of a single chip. Roughly 30% of the PFTCS on the stamp is transferred to the PDMS microvalve and this 30% is split evenly between the microvalve and glass substrate after the final bonding step.

As an additional demonstration for this selective bonding technique, the stretchable microdevice was fabricated and tested by observing the assembly and quality of the designed 3D structures. To fabricate the stretchable device shown in Fig. SI8, thin PDMS was unidirectionally stretched 15%, masked, PFTCS deposited, and then bonded to a PDMS ribbon after alignment with the PFTCS pattern before relaxation. Multiple arched shaped 3D structures were formed successfully without any heavy microfabrication procedures when stretching of thin PDMS substrate was relaxed.

### Stability and longevity tests

**Figure 5:**
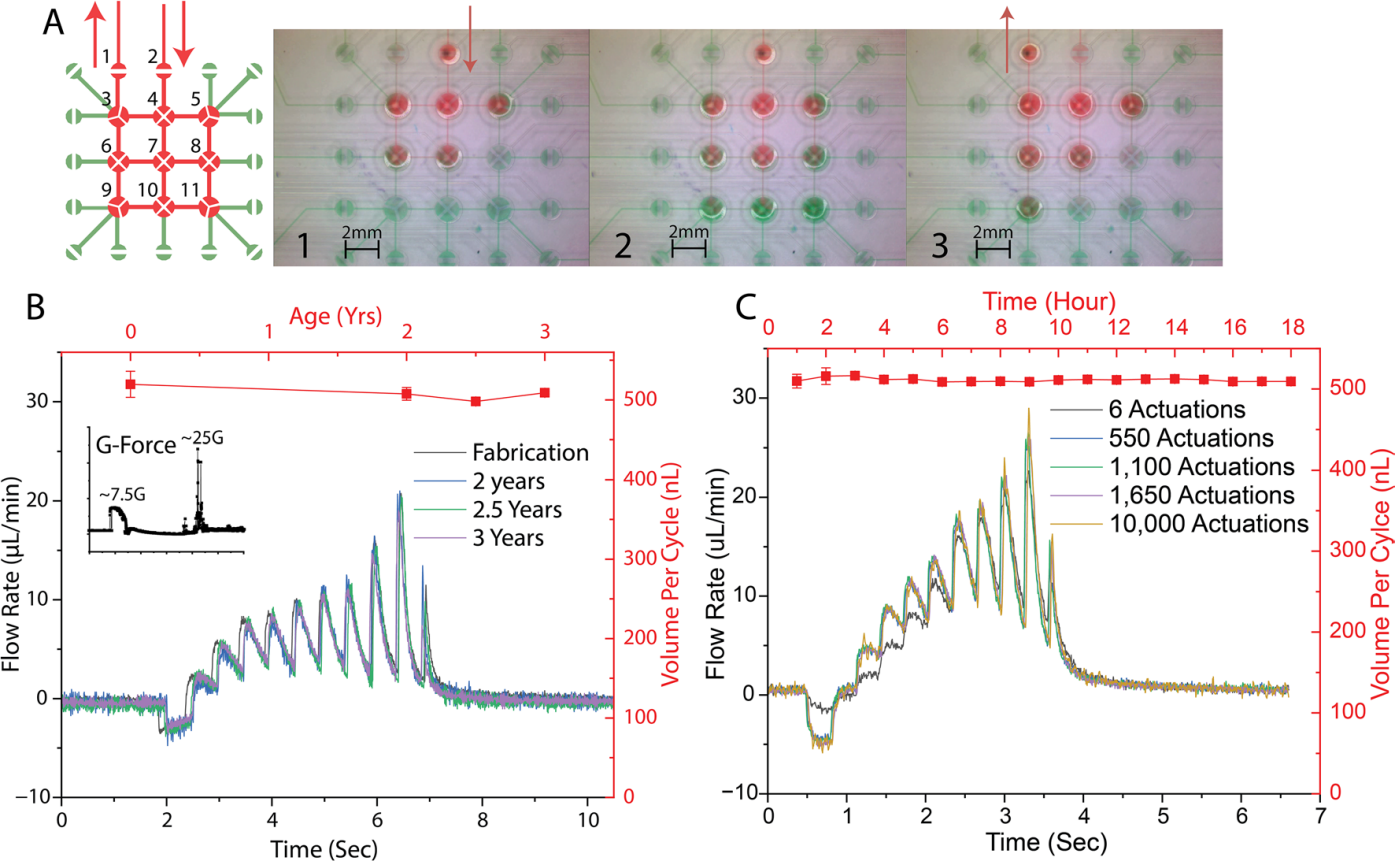
Different long-term stability tests for the presented fabrication method. (**A**) Diagram of example pumping sequence that pulls in fluid and then pushes it out to where it is needed. The numbers in the diagram designate valve numbers for the actuation sequence given in supplemental information. (**B**) Comparison of flow rate profile from a complex microvalve based device fabricated using this PFTCS based method on the date of fabrication and 3 years later. The inset plot shows a minute-long g-Force profile this chip experienced during a rocket launch and crash between the time of the two flow rate plots. The launch had a sustained 7.5 g acceleration applied to the chip and during the crash the acceleration was as high as 25 g. This shows the longevity capabilities and resilience of this microcontact printing method. (**C**) Comparison of the performance of the same type of device with constant use over thousands of open and closing operations. The specified actuations correspond to a single valve in the sequence meaning each of the 11 valves experienced that many actuations.

Figure 5 shows a larger scale PMA with 25 microvalves that was fabricated using the optimized parameters to perform complex fluidic manipulation. This PMA is designed to be a pumping component for a full fluidic processor. If combined with a detection system, the combination can be used for the detection of life signatures in extra-terrestrial samples in-situ by leveraging the long-term stability offered with the presented fabrication method. For example, analysis of ice samples from the moons of Saturn and Jupiter can reveal signs of life in our solar system^35^ and a PMA fabricated with the presented fabrication method has the capability to not only preform the necessary processing but still be functional after years of storage during flight.

Thus, it is important to verify the stability of this component after both long-term actuations, simulated rocket launch, and long-term storage. Figure 5A shows the PMA design and snapshots of the pumping sequence with color dyes used in the stability testing. The numbering in the cartoon designates valve numbers for the actuation sequence given in the supplemental information (Table SI2). The sequence started by opening one of the perimeter two-way valves and then in sequence opening the interior four-way valves until all are open shown in Fig. 5A1. Once all the four-way valves are open (Fig. 5A2), the inlet two-way valve is closed, and then an outlet two-way valve is opened. Last, the interior four-way valves are closed in sequence to push out the fluid as presented in Fig. 5A3. This pumping sequence were repeated for the following stability tests under various conditions. Figure 5B and C show the flow rate of this sequence after various storage, force, and actuation conditions, as well as the average volume per cycle of the pumping sequence in each case. Figure 5B shows that this method is stable with respect to long rest times, > 3 years, as the flow profile is identical after fabrication and three years later. In addition, the inset graph in Fig. 5B shows a g-Force plot experienced by that particular chip during a test rocket flight and crash between the time of the two flow rate measurements. The average g-force during launch was about 7.5 g matching what slightly higher expected during typical launches, 3–6 g^36^, and the chip was able to withstand forces up to 25 g during the crash. During the descent and crash, the PMA also was subjected to vibration at 7.5 Hz for approximately 10 s. The resilience of the PMA to these conditions proves that the PFTCS selective bonding method is stable during and after a rocket launch. Other chips tested after long rest times (> 3 years) show some slow response times when initially reopening valves. After a few priming actuations of ~ 10 times, the PMA returns to its usual operating conditions. This long-term storage demonstrates the resilience of the PDMS-glass bond around the microvalve. As this is an irreversible bond, bond failure is not anticipated; however, the PDMS-glass bond around the microvalve is subjected to relatively high pressures for short periods of time when the valve closes. During the observation, there are no evidence of failure or alterations in this region, indicating the continued strength of the bond. In addition, the high usage results presented in Fig. 5C show the stability and consistency of the PMA despite repetitive tasks and sequences. Each of the measured points has an identical plot regardless of number of actuations. After 18 h of constant actuation of all microvalves in the PMA, there are almost no difference on PMA performance. These results show that the PFTCS assisted selective bonding method can be trusted for space missions that have long wait times and high number of cycle operations without risk of failure on microfluidic operations. Further characterizations are underway to understand thermal profiles and PMA actuation performance under zero g.

## Conclusion

To support long-term space mission using a programmable microvalve array, a selective PDMS bonding procedure using PFTCS micro-contact printing has been characterized and demonstrated for the first time. The stamp fabrication, PFTCS deposition, and use of the stamps were characterized in detail, and the primary parameters such as temperature, pressure, and loading condition for deposition and stamp were identified to achieve selective bonding of PDMS on various substrates. In particular, we determined the dominant parameters for both deposition, high vacuum level, and stamping, high temperature. The other parameters tested, source temperature and time during deposition and stamping time and material during transfer, were not influential with respect to background noise in the data. Using the protocol detailed in this manuscript, PFTCS was deposited on PDMS stamps which were used to transfer the PFTCS to both glass and PDMS to demonstrate microvalve array and a stretchable 3D ribbon device. In addition, this fabrication method is stable over long time periods, high number of actuations, and exposure to high g-forces making it useful for long-term space missions without any alteration of microvalve functionality. Furthermore, the presented selective bonding technique is easily scalable to larger devices with high density microvalve array. A more complex PMA than presented here would be able to store and dilute reagents, mix and label samples, and deliver the labeled sample to a detection system, all with the long-term stability required for missions in the outer solar system. That hypothetical PMA would be able to be used for many different mission profiles since its programmability allows for protocol changes without changing the physical design of the chip. Additional testing will be beneficial particularly for thermocycling to understand thermal expansion-induced thermal stress and comprehensive vibration to evaluate mechanical stability. Despite preliminary results for these types of tests are promising, more extensive environmental testing is still necessary to enhance the technological readiness level (TRL). In addition, the presented fabrication method can be improved through the use of photoresist-based masking of the PDMS stamp. This would greatly increase the possible resolution of this method to create more intricate surface treatment patterns.

## Supplementary Information

Supplementary Information.

## Data Availability

The datasets generated and analyzed during this work are not publicly available due to patent application consideration but are available from the corresponding author on reasonable request.
