# Supplementary material for: Microvalve array fabrication using selective PDMS (polydimethylsiloxane) bonding through Perfluorooctyl-trichlorosilane passivation for long-term space exploration
Source: Sci Rep. 2022 Jul 20;12:12398. doi: 10.1038/s41598-022-16574-9 (PMC9300634; doi:10.1038/s41598-022-16574-9)
Supplement: Supplementary file 1 — Supplementary Information. [file 41598_2022_16574_MOESM1_ESM.pdf]

Supplemental information for
Microvalve array fabrication using selective PDMS (polydimethylsiloxane) bonding through Perfluorooctyl-trichlorosilane passivation for long-term space exploration

Zachary Estlack and Jungkyu (Jay) Kim*

Department of Mechanical Engineering, University of Utah, Salt Lake City, UT 84112

* Address correspondence to: Jungkyu (Jay) Kim

Department of Mechanical Engineering

University of Utah

Salt Lake City, UT 84112

Phone: (801)581-6743

E-mail: jkim@mech.utah.edu

Preliminary Plasma Survivability Testing

An oxygen plasma test was done to ensure the survival of the PFTCS surface after plasma etching. Stamps with PFTCS deposited were placed into an oxygen plasma chamber and exposed to plasma for 30 seconds. The thickness of the PFTCS was measured before and after the exposure and a change of only ~10% was seen.

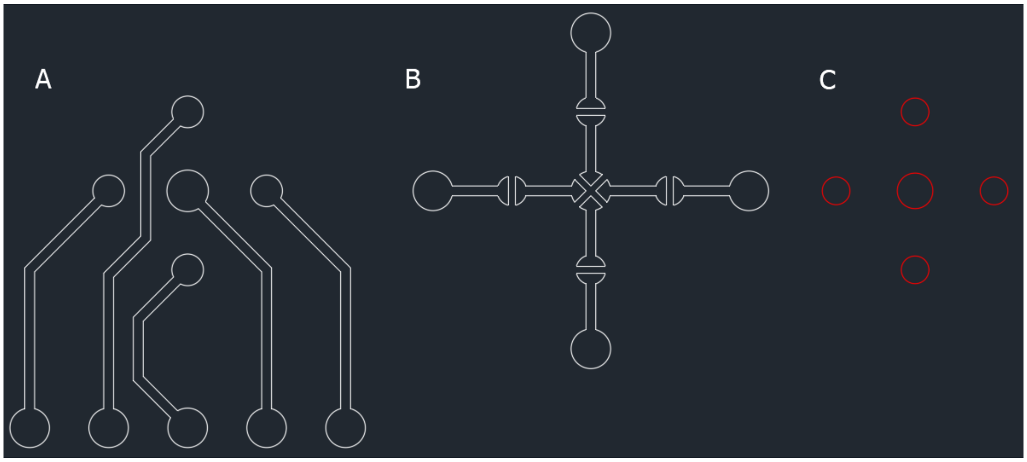

Figure SI1: Designs used for the gate structure valves. A) Pneumatic Layer, B) Fluidic Layer showing the four two-way valves and single four-way valve, C) Stamp mask

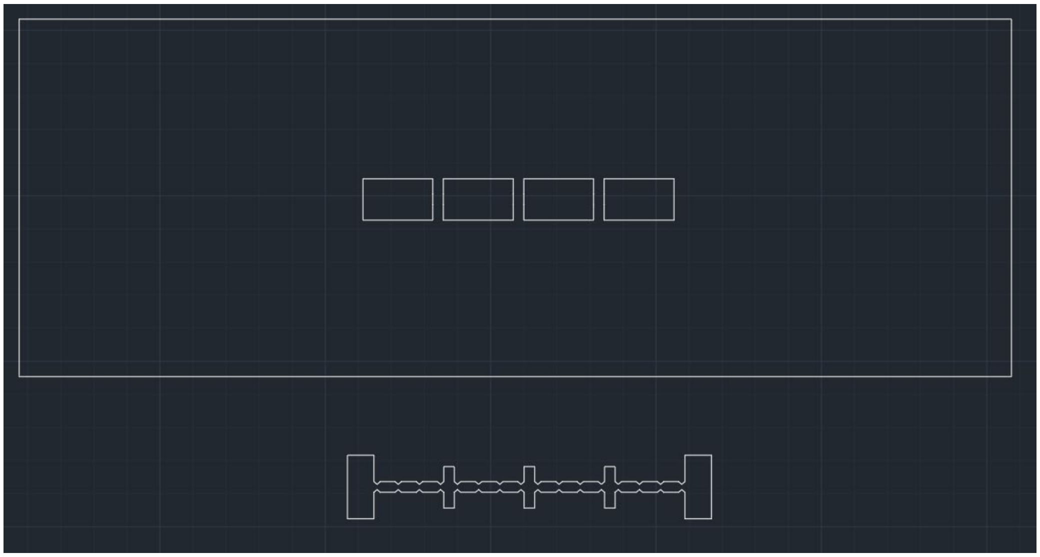

Figure SI2: Stretchable device mask (top) and PDMS ribbon (bottom)

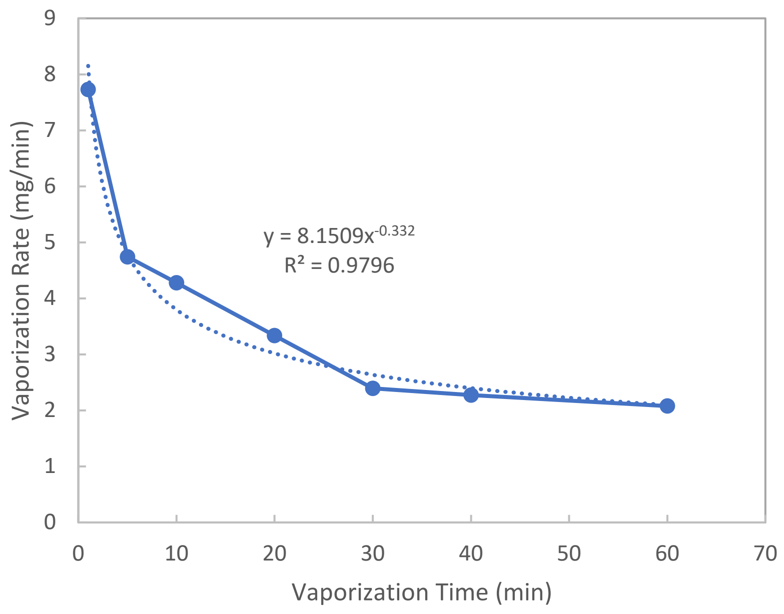

Figure SI3: Characterization of the vaporization rate of PFTCS under -80 kPa vacuum

The large-scale PMA was secured in an enclosure to prevent cracking of the backing glass and secured to a bulkhead in the main structure of the rocket, in line with the main axis. The launch was performed with a Cessaroni L1395-BS (Cesaroni; Canada) motor capable of generating 313 pounds of thrust and the rocket reached a height of 13,000 feet. A video of the launch is provided by Raider Aerospace at https://www.youtube.com/watch?v=v3j7MeSm4XA.

Table SI1: Rocket Launch Parameters

Parameter	Value
Motor	Cessaroni L1395-BS
Thrust	313 pds
Height	13,000 ft
Max launch G	7.5G
Max Overall G	25G
Flight Time	~40 sec

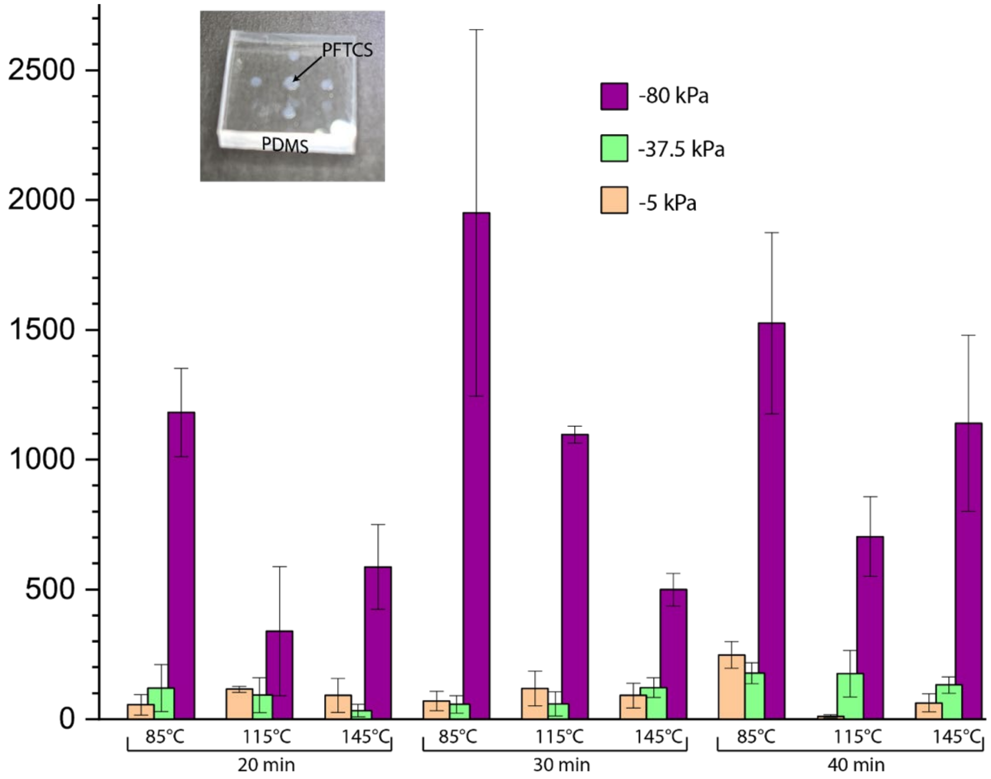

Figure SI4: Experimental Characterization of the deposition of PFTCS onto a PDMS stamp; Inset: Picture of a stamp after deposition.

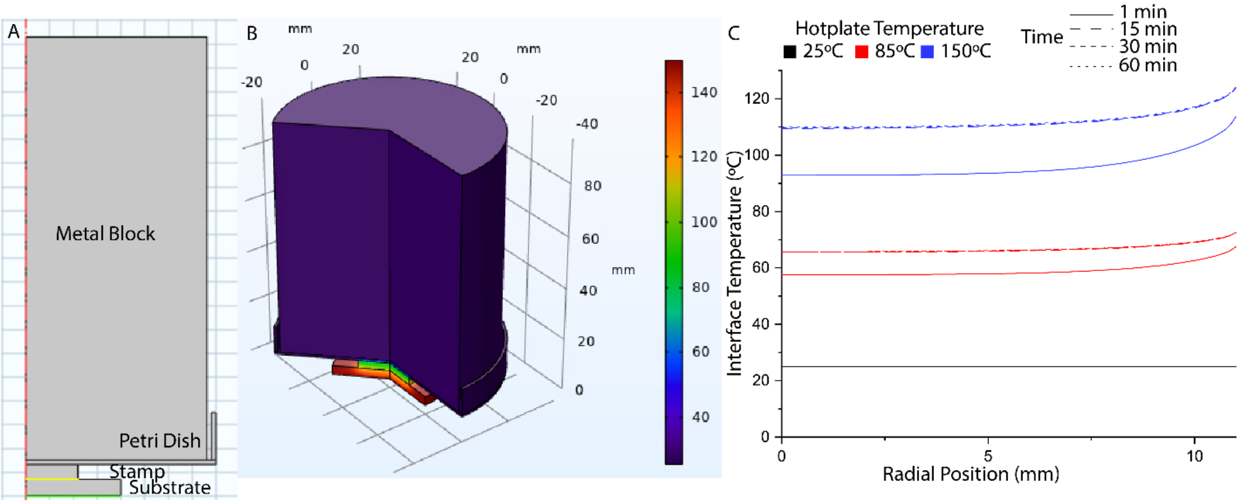

Figure SI5:2D axisymmetric heat transfer simulation of the PDMS stamping setup. A) Setup for the simulation. The yellow line marks the interface where the PFTCS transfer occurs, and the green line is set to a constant temperature. All other exterior boundaries are set to be open. B) 3D view of the stamping setup and the temperatures after 60 minutes with a bottom temperature of 150°C and a PDMS substrate. C) Temperature measurement at the stamp interface with the setup described.

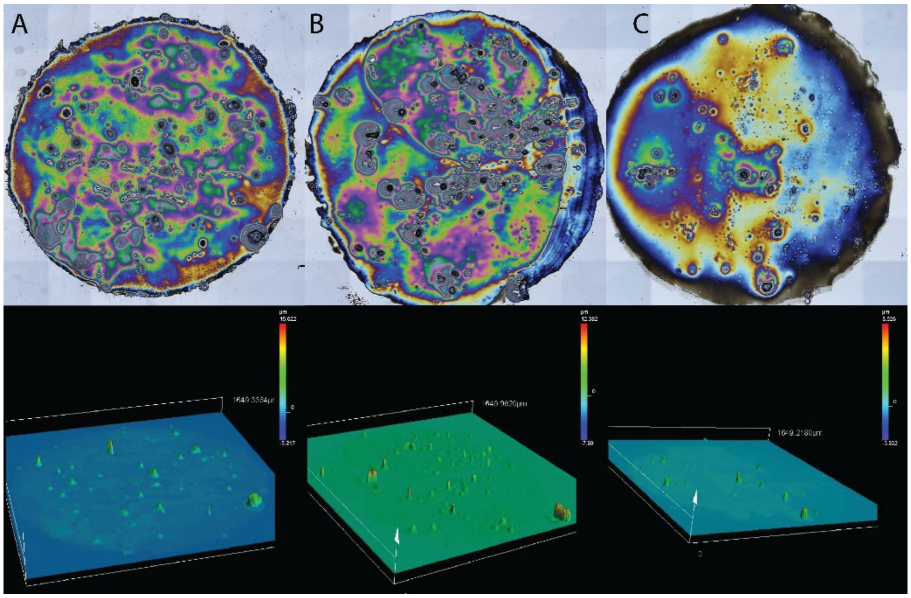

Figure SI6: Image of PFTCS stamped onto glass showing the quality and uniformity of the transfer after 30 minutes at A) 25ºC, B) 85ºC, C) 150ºC. Top is the color view of the surface and the bottom is a 3D view generated by the microscope.

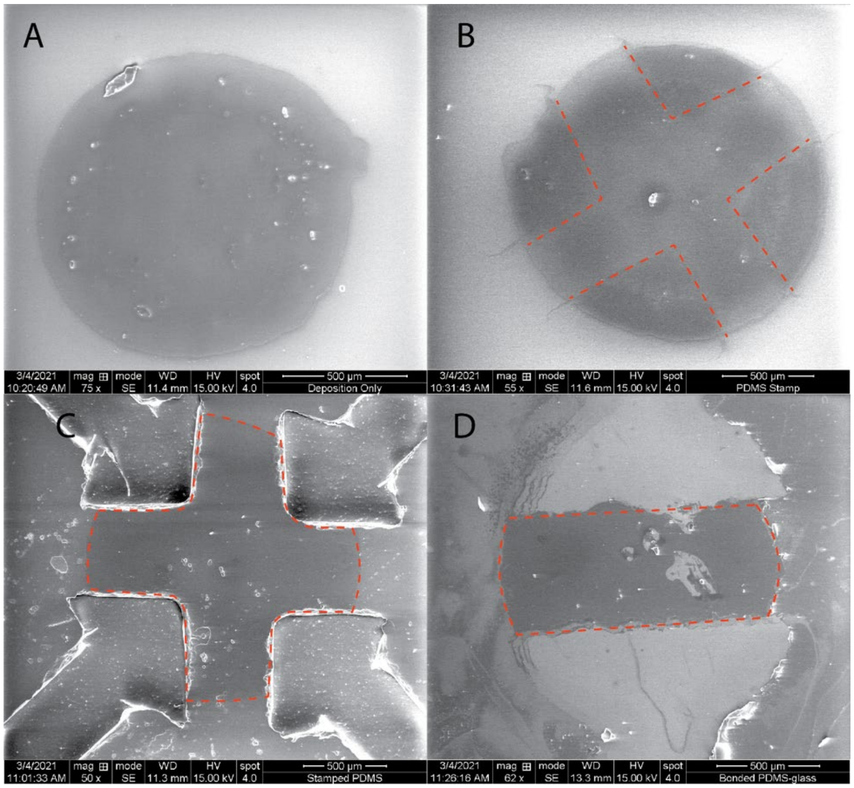

Figure SI7: Scanning electron microscope images of: A) PFTCS deposition onto a stamp, B) Stamp after cross shaped transfer to a four-way valve (dotted line shows the transfer region), C) Four-way PDMS gate after being stamped on (dotted line highlighting the transferred region), and D) Glass surface after final bonding and PDMS removal showing the presence of the PFTCS on its surface despite the PFTCS being stamped on the two-way gate (dotted line highlighting the region with PFTCS).

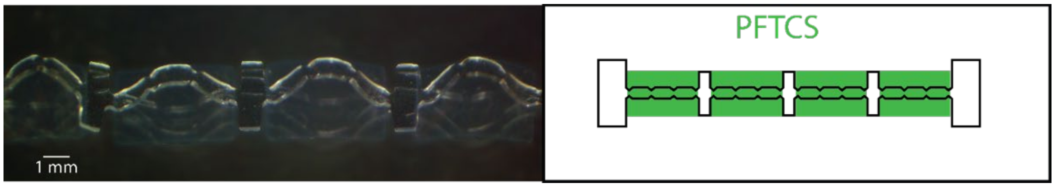

Figure SI8: Stretchable device anchored intermittently. The cartoon on the right shows where PFTCS was deposited prior to bonding.

Table SI2: PMA chip actuation sequence for functional testing

Operation	1st	2nd	3rd	4th	5th	6th	7th	8th	9th	10th	11th
Opening	2	4	5	3	6	7	8	9	10	11	1
Closing	2	11	10	8	9	7	5	6	4	3	1
